# Associations of Physical Activity, Sedentary Behaviour, and Sleep with Somatotype Components and a Body Shape Index in Young Adults

**DOI:** 10.3390/jfmk11030367

**Published:** 2026-09-10

**Authors:** Agnieszka Wasiluk, Robert Wilczewski, Katja Zdešar Kotnik, Tatjana Robič Pikel

**Affiliations:** 1 Department of Health Promotion, Faculty of Physical Education and Health in Biala Podlaska, Jozef Pilsudski University of Physical Education in Warsaw, 00-968 Warsaw, Poland; 2 Department of Biology, Biotechnical Faculty, University of Ljubljana, 1000 Ljubljana, Slovenia

**Keywords:** anthropometry, Heath–Carter method, lifestyle behaviours, waist circumference, university students

## Abstract

**Background**: The Body Shape Index (ABSI) and somatotype components represent different approaches to assessing body morphology. This study aimed to examine the associations between physical activity, sedentary behaviour, sleep duration, ABSI, and somatotype components in young adults. **Methods**: A cross-sectional study was conducted among 371 university students from Poland and Slovenia (202 men and 169 women; mean age: 21.46 ± 1.07 years). ABSI and somatotype components were determined based on anthropometric measurements. Data on physical activity, sedentary behaviour, and sleep duration were obtained using a seven-day activity record. Associations were assessed using Spearman’s rank correlation coefficients and multiple linear regression models adjusted for sex, age, and country of origin. **Results**: Weak negative correlations were observed between walking time and ABSI (r_s_ = −0.135; *p* < 0.01) and between sleep duration and mesomorphy (r_s_ = −0.111; *p* < 0.05). Additional weak associations were identified in analyses conducted separately for women and men. After adjustment for potential confounders, walking time was positively associated with endomorphy (β = 0.158; *p* = 0.009) and mesomorphy (β = 0.117; *p* = 0.041), and negatively associated with ectomorphy (β = −0.192; *p* = 0.002). All significant associations were characterized by small effect sizes. No significant associations were found for ABSI in the primary adjusted model for the total sample. **Conclusions**: Despite the small magnitude of the observed associations, different patterns of associations were observed for ABSI and the somatotype profile, reflected by the combined pattern of its endomorphic, mesomorphic, and ectomorphic components. However, the identified associations were few in number and weak in strength; therefore, further research is needed to better explain their significance in young adult populations.

## 1. Introduction

Physical activity, sedentary behaviour, and sleep are interrelated lifestyle behaviours that collectively influence health and overall human functioning [1]. Regular physical activity is associated with numerous health benefits, whereas insufficient physical activity and prolonged sedentary behaviour are linked to an increased risk of adverse health outcomes and premature mortality [2,3]. Sleep is another important component of a healthy lifestyle, and its adequate duration and quality play a crucial role in maintaining both physical and mental health [1,4]. These behaviours are of particular importance during early adulthood, including the university years, when long-term health-related habits are established. Previous studies have shown that the transition to university is often accompanied by decreased physical activity levels, poorer adherence to 24-h movement guidelines, and increased sleep disturbances [5]. Such behaviours may affect not only health status but also body composition and body morphology. Indeed, physical activity levels and sleep quality have been shown to be associated with selected body composition parameters and anthropometric indicators in young adults [6].

Assessment of body morphology is an important component of research on the health of young adults [7]. The most commonly used anthropometric indicator remains the body mass index (BMI), which, due to its simplicity of calculation and interpretation, is widely applied in both epidemiological research and clinical practice [8]. Despite its widespread use, BMI does not account for body composition or fat distribution, meaning that individuals with identical BMI values may differ substantially in terms of fat mass and fat-free mass proportions [8,9]. These limitations are particularly evident among physically active young adults, in whom higher BMI values may reflect greater muscle mass rather than excess adiposity [10]. Consequently, increasing attention has been directed toward alternative anthropometric indices that consider not only body size but also fat distribution. Particular interest has focused on indicators assessing central adiposity, which is recognized as an important risk factor for numerous chronic diseases [7,11].

One such indicator is the A Body Shape Index (ABSI), introduced by Krakauer and Krakauer [9] as a measure incorporating waist circumference relative to height and body mass. Unlike BMI, ABSI provides information on waist circumference relative to body size, which may be relevant to health risk assessment [12]. Subsequent studies have demonstrated associations between ABSI and the risk of cardiovascular diseases, hypertension, metabolic disorders, and all-cause mortality, as evidenced by both observational studies and systematic reviews [13,14,15,16]. ABSI has also been increasingly used in research examining lifestyle factors, physical activity, and other health-related behaviours [17,18]. For example, Li et al. [19] reported independent as well as combined associations of physical activity and ABSI with sleep disturbances. Despite the growing body of research on ABSI, knowledge regarding the associations between this index and everyday health behaviours in young adults remains limited [18,19].

An additional approach to the assessment of body morphology is the Heath–Carter somatotype, which describes human physique using three components: endomorphy, mesomorphy, and ectomorphy [20]. Endomorphy reflects the relative level of body fatness, and mesomorphy characterizes musculoskeletal development, whereas ectomorphy represents the linearity of the physique [20]. The somatotype is widely used in biological anthropology, sport sciences, and health research as a tool for the comprehensive assessment of body morphology [21]. Previous studies have demonstrated associations between somatotype components and body composition, risk factors for non-communicable diseases, and selected health indicators [22,23]. Relationships between somatotype and cardiorespiratory fitness have also been reported [24]. Furthermore, available evidence suggests that physical activity levels may be associated with somatotype components, particularly mesomorphy [25,26]. A limited number of studies have also considered other lifestyle factors, such as sedentary behaviour, in analyses of body morphology and health [27]. Nevertheless, little is known about the associations between body morphology indicators, such as ABSI and somatotype, and everyday health behaviours, including physical activity, sedentary behaviour, and sleep.

Previous research has primarily focused on individual body morphology indicators or selected lifestyle behaviours. In the case of ABSI, studies have mainly examined its associations with health risk, cardiovascular diseases, and mortality [14,16]. In contrast, research on somatotype has largely focused on body composition, physical fitness, and the characteristics of athletic populations [21,24,26]. Although some studies have investigated the associations of either somatotype or ABSI with selected health behaviours, the simultaneous assessment of physical activity, sedentary behaviour, and sleep in relation to both of these indicators remains poorly understood, particularly among young adults [19,27]. Based on the available evidence, it may generally be expected that higher levels of physical activity, lower sedentary behaviour, and adequate sleep duration are associated with differences in body morphology indicators, including ABSI and somatotype components. However, the limited number of studies and the inconsistency of previous findings do not allow the formulation of specific hypotheses regarding the strength or direction of these relationships. Therefore, the present study was designed as an exploratory investigation aimed at examining the associations between physical activity, sedentary behaviour, sleep duration, and body morphology, assessed using somatotype components and A Body Shape Index (ABSI), in young adults.

## 2. Materials and Methods

### 2.1. Participants and Study Procedure

This cross-sectional study was conducted among undergraduate students from Poland and Slovenia. A total of 371 participants were included in the analyses, comprising 202 men and 169 women. No a priori sample-size calculation was performed because the final sample size was determined by the number of eligible students from the participating academic programmes who were available during the study period and voluntarily agreed to participate. A sensitivity analysis based on the achieved sample size (*n* = 371), eight independent variables, α = 0.05, and 80% statistical power indicated that the regression analyses were capable of detecting an effect size of approximately Cohen’s f^2^ = 0.04. The mean age of the participants was 21.46 ± 1.07 years. The Polish sample consisted of Physical Education students enrolled at the Faculty of Physical Education and Health in Biała Podlaska. The Slovenian sample included students of the University of Ljubljana studying biology, food science and nutrition, and double-subject teacher education programmes combining biology with chemistry or biology with home economics. At both study sites, students from the participating academic programmes who were available during the study period were invited to participate. The Slovenian participants were analysed as a single group because the individual academic programmes included small and uneven numbers of participants, particularly when stratified by sex. The inclusion of participants from two countries was intended to increase the diversity of the study sample in terms of lifestyle behaviours and body morphology characteristics while maintaining a similar age range and educational status. Academic programme was not included as a separate covariate in the regression models. Country of origin was included as a control variable to account for overall differences between the Polish and Slovenian samples. However, because country and academic profile were closely linked in the present sample, their potential effects could not be fully disentangled. Participation in the study was voluntary, and all participants were informed about the purpose and procedures of the research. Only individuals with complete anthropometric data and information regarding physical activity, sedentary behaviour, and sleep duration were included in the analyses. The study received approval from the relevant ethics committees at both study sites and was conducted in accordance with the principles of the Declaration of Helsinki.

### 2.2. Anthropometric Measurements and Assessment of Body Morphology

Anthropometric measurements were performed by trained researchers at each study site using the same predefined standardized measurement protocol [28]. The anthropometric measurements were performed by experienced researchers with extensive professional experience in anthropometric assessment. The first author participated directly in the anthropometric measurements at both study sites, supporting consistency in the application of the measurement protocol. The same measurement equipment and measurement sequence were used at both sites, and measurements were performed under comparable conditions. Body height was measured to the nearest 0.1 cm using a portable stadiometer, while body mass was assessed to the nearest 0.1 kg using a calibrated electronic scale (Tanita MC-780MA, Tokyo, Japan). All measurements were taken with participants barefoot and wearing light sports clothing. Waist circumference was measured midway between the lower margin of the last rib and the upper border of the iliac crest, whereas hip circumference was measured at the level of the maximum protrusion of the buttocks. In addition, flexed arm circumference and calf circumference were measured. All circumferences were assessed to the nearest 0.1 cm using a non-elastic anthropometric tape (Cescorf Equipamentos, Tristeza–Porto Alegre, Brazil). Skinfold thicknesses were measured using a Harpenden skinfold caliper (John Bull British Indicators Ltd., London, UK) to the nearest 0.2 mm, with each skinfold measured three times. Biepicondylar breadths of the humerus and femur were assessed using a Martin anthropometer (small sliding anthropometer, RealMet Institute, Barcelona, Spain) to the nearest 0.1 cm. Based on the available repeated skinfold measurements, the technical error of measurement (TEM) was 0.86 mm for the triceps, 1.18 mm for the subscapular, 0.62 mm for the supraspinal, and 1.09 mm for the medial calf skinfold.

Based on the obtained measurements, body mass index (BMI) was calculated as body mass in kilograms divided by the square of body height in meters (kg/m^2^) [29]. The waist-to-hip ratio (WHR) was calculated as the ratio of waist circumference to hip circumference [30], whereas the waist-to-height ratio (WHtR) was calculated as the ratio of waist circumference to body height [31]. A Body Shape Index (ABSI) was calculated according to the equation proposed by Krakauer and Krakauer [9]: ABSI = WC/(BMI^(2/3)^ × height^(1/2)^), where waist circumference (WC) and height were expressed in meters and BMI in kg/m^2^. Raw ABSI values were used as a continuous body-shape measure and were not interpreted according to risk categories. Age and sex were included as covariates in the regression models to account for their potential influence on ABSI. Somatotype was determined using the anthropometric Heath–Carter method [20]. Endomorphy was calculated from the sum of the triceps, subscapular, and supraspinal skinfolds, adjusted for body height according to the Heath–Carter method. Mesomorphy was calculated from humerus and femur breadths, arm and calf circumferences corrected for the corresponding skinfold thickness, and body height. Ectomorphy was determined from the height–weight ratio. The standard Heath–Carter equations were applied identically to women and men. Calculations were performed using spreadsheet-based formulas according to the procedures described in the original methods [9,20], without modification of the original equations.

### 2.3. Assessment of Physical Activity, Sedentary Behaviour, and Sleep Duration

Information on physical activity, sedentary behaviour, and sleep duration was collected using a self-reported seven-day activity record form developed by the authors. The form was not independently validated; therefore, instrument-specific validity and reliability estimates are not available. The form was based on physical activity categories corresponding to those used in the International Physical Activity Questionnaire (IPAQ) and was designed to facilitate daily recording by the participants. The form enabled participants to record the amount of time spent each day walking, engaging in moderate- and vigorous-intensity physical activity, sitting, and sleeping. To facilitate the distinction between moderate- and vigorous-intensity physical activity, participants were instructed to use the talk test: activity during which conversation could be maintained comfortably was considered moderate, whereas activity causing breathlessness that made conversation difficult was considered vigorous. Sedentary time included time spent sitting during study-related activities, leisure time, and transportation. Sleep duration was recorded as the total number of hours slept each day. Completed records were checked by the researchers for completeness, and all participants provided complete records for all seven consecutive days. Daily values were summed across the seven-day period. Based on the collected data, weekly time spent walking and engaging in moderate- and vigorous-intensity physical activity was calculated and expressed in minutes per week. In addition, total weekly sedentary time and total sleep duration were determined and expressed in hours per week. All variables related to physical activity, sedentary behaviour, and sleep were analysed as continuous variables. This approach allowed the full variability of the data to be retained and avoided the loss of information associated with categorising participants according to physical activity levels.

### 2.4. Statistical Analysis

Statistical analyses began with the calculation of descriptive statistics. The normality of distributions was assessed using the Shapiro–Wilk test. Variables with approximately normal distributions were presented as means and standard deviations (mean ± SD). Due to the skewed distribution of variables related to physical activity, sedentary behaviour, and sleep, these data were presented as medians and interquartile ranges (IQR). Because some of the analysed variables did not meet the assumption of normality, Spearman’s rank correlation coefficients were calculated to assess the associations between physical activity, sedentary behaviour, sleep, and ABSI as well as somatotype components. Analyses for the entire sample were conducted to evaluate overall associations between lifestyle behaviours and body morphology indicators in the study population. In addition to the analyses performed for the entire sample, correlation analyses were conducted separately for women and men to examine whether the observed associations were generally consistent across both sex-specific subgroups. These analyses were exploratory in nature and were conducted to identify potential associations between the analysed variables rather than to compare women and men. No formal interaction tests between sex and behaviour were performed; therefore, differences in statistical significance between the sex-stratified analyses were not interpreted as evidence of statistically significant differences between women and men. Given the exploratory nature of the analyses, no formal adjustment for multiple comparisons was applied. Accordingly, the reported *p*-values should be regarded as nominal and interpreted cautiously, with consideration of the magnitude and consistency of the observed associations rather than statistical significance alone. Correlation results were interpreted cautiously, considering not only statistical significance but also effect sizes and the results of the multiple regression analyses. Independent associations between physical activity, sedentary behaviour, sleep, and ABSI as well as somatotype components were assessed using multiple linear regression. Separate regression models were constructed for ABSI, endomorphy, mesomorphy, and ectomorphy as dependent variables. The three somatotype components were analyzed separately to examine whether individual dimensions of physique showed distinct associations with movement behaviours. However, because endomorphy, mesomorphy, and ectomorphy jointly define the overall somatotype, associations across the three components were interpreted collectively as patterns of somatotype variation rather than as independent morphological characteristics. Walking time, moderate-intensity physical activity, vigorous-intensity physical activity, sedentary time, and sleep duration were entered into the models as independent variables. All models were adjusted for sex, age, and country of origin. To examine whether the observed associations were consistent across the two study populations, additional regression analyses were conducted separately for the Polish and Slovenian participants. These country-stratified models included the same lifestyle behaviour variables as the main models and were adjusted for sex and age. Sex and country of origin were included as dichotomous categorical variables. Sex was coded as 1 = male and 2 = female, whereas country of origin was coded as 1 = Slovenia and 2 = Poland. Before model interpretation, multicollinearity was assessed using the variance inflation factor (VIF), and influential observations were examined. No evidence of multicollinearity was detected among the independent variables (all VIF values < 2). To facilitate interpretation of the regression coefficients, walking time, moderate-intensity physical activity, and vigorous-intensity physical activity were expressed per 100 min per week. ABSI values were multiplied by 1000 solely to improve the readability of the regression coefficients. Model results were reported as unstandardized coefficients (B), standard errors (SE), standardized regression coefficients (β), 95% confidence intervals (95% CI), coefficients of determination (R^2^), and *p*-values. Statistical significance was set at *p* < 0.05. All statistical analyses were performed using Statistica 13.0 software (StatSoft Polska, Kraków, Poland).

## 3. Results

The anthropometric characteristics of the participants, including body dimensions, circumferences, skeletal breadths, and skinfold thicknesses, are presented in Table 1.

Table 2 summarizes age, body morphology indices, somatotype components, physical activity, sedentary behaviour, and sleep duration for the total sample and separately for women and men. Sex-specific values are presented for descriptive purposes only.

The results of Spearman’s rank correlation analyses for the entire study sample, as well as for men and women separately, are presented in Table 3. Significant but weak negative correlations were observed between walking time and ABSI (r_s_ = −0.135; *p* < 0.01) and between sleep duration and mesomorphy (r_s_ = −0.111; *p* < 0.05). The remaining correlation coefficients did not reach statistical significance. Among men, only one statistically significant association of small magnitude was identified. Sedentary time was negatively associated with ectomorphy (r_s_ = −0.138; *p* < 0.05). No significant associations were observed between the remaining variables. Among women, several statistically significant associations of small magnitude were observed. All significant associations were characterized by small correlation coefficients. Vigorous-intensity physical activity was negatively correlated with ABSI (r_s_ = −0.162; *p* < 0.05). In addition, moderate-intensity physical activity was negatively correlated with ectomorphy (r_s_ = −0.154; *p* < 0.05). Sleep duration was negatively correlated with mesomorphy (r_s_ = −0.194; *p* < 0.05) and positively correlated with ectomorphy (r_s_ = 0.166; *p* < 0.05). No statistically significant associations were found for the remaining variables.

To assess the independent associations of physical activity, sedentary behaviour, and sleep with ABSI and somatotype components, multiple linear regression analyses were performed (Table 4). After adjustment for sex, age, and country of origin, no significant associations were observed between the analysed behaviours and ABSI. Sex was the only covariate significantly associated with ABSI. Among the somatotype components, walking time was the only variable consistently associated with body morphology. Higher walking time was positively associated with both endomorphy (β = 0.158; *p* = 0.009) and mesomorphy (β = 0.117; *p* = 0.041), and negatively associated with ectomorphy (β = −0.192; *p* = 0.002). In contrast, no significant associations were found between moderate- or vigorous-intensity physical activity, sedentary time, or sleep duration and any of the analysed somatotype components. Among the control variables, sex was significantly associated with ABSI, endomorphy, and mesomorphy, whereas country of origin showed significant associations with all somatotype components. Age was not significantly associated with any of the dependent variables in the analysed models.

In additional country-stratified regression analyses, several statistically significant associations were observed within the Polish and Slovenian samples (Table 5). Among Slovenian participants, walking time was positively associated with endomorphy (β = 0.221; *p* = 0.019) and negatively associated with ectomorphy (β = −0.214; *p* = 0.032), while moderate-intensity physical activity was negatively associated with ABSI (β = −0.269; *p* = 0.006). Among Polish participants, moderate-intensity physical activity was positively associated with endomorphy (β = 0.137; *p* = 0.020) and negatively associated with ectomorphy (β = −0.125; *p* = 0.046), while sleep duration was negatively associated with mesomorphy (β = −0.129; *p* = 0.020).

## 4. Discussion

In the primary adjusted analysis of the total sample, no significant independent associations were observed for ABSI, whereas significant associations were identified for the somatotype components. Greater walking time was associated with higher endomorphy and mesomorphy and lower ectomorphy. However, given the limited number of significant associations and their small effect sizes, these findings should be interpreted with caution.

In contrast to the somatotype components, ABSI was not significantly associated with any of the analysed health behaviours in the primary adjusted analysis of the total sample. Previous studies on ABSI have focused primarily on its role as an indicator of health risk, including the risk of cardiovascular diseases, metabolic syndrome, sarcopenia, and all-cause mortality [32,33,34]. Only in recent years has this index been increasingly applied in studies examining health behaviours and lifestyle factors [17,18]. The lack of observed associations in the present study may be partly related to the characteristics of the study population. The participants were young adults whose ABSI values showed relatively limited variability. In contrast to many previous studies conducted among middle-aged and older adults or populations with elevated health risks [32,33,34], the present sample consisted of young and relatively healthy individuals. It is therefore possible that the restricted variability of ABSI values within the study sample reduced statistical sensitivity and limited the likelihood of detecting associations between this index and the analysed health behaviours. The findings of the present study are partly consistent with previous observations reported among young adults. Guerrero-Agenjo et al. [18] likewise found no significant associations between physical activity levels and ABSI values in a sample of university students. In contrast, Li et al. [19] reported that higher ABSI values were associated with an increased likelihood of sleep disorders among older adults with cardiometabolic multimorbidity. However, it should be noted that their study was conducted among older adults with cardiometabolic multimorbidity, which differs substantially from the population examined in the present study. These differences suggest that the associations between ABSI and health behaviours may depend on population characteristics, including age and overall health status.

In contrast to ABSI, adjusted associations were observed between walking time and the somatotype components. Greater walking time was independently associated with higher values of endomorphy and mesomorphy and lower values of ectomorphy. Considered jointly, these associations indicate a somatotype pattern characterized by relatively higher endomorphic and mesomorphic components and a lower ectomorphic component, rather than three independent morphological associations. However, it should be emphasized that the observed associations were weak. Moreover, the physiological interpretation of this combined pattern is not straightforward, and the present cross-sectional findings do not provide a basis for attributing a specific physiological meaning to these associations. Somatotype reflects overall body morphology by incorporating relative adiposity, musculoskeletal development, and physique linearity [20]. Unlike ABSI, which reflects waist circumference relative to body size, somatotype provides a more comprehensive description of overall body morphology. It is possible that differences in the type of information captured by these two indicators contributed to the observation of associations for somatotype components but not for ABSI. These findings indicate different patterns of associations for ABSI and somatotype components within the present sample; however, the study was not designed to compare the validity, predictive ability, or superiority of these measures.

Available evidence regarding the associations between physical activity and somatotype remains limited. Pereira et al. [25] reported that more physically active individuals were characterized by higher mesomorphy, whereas Esteve-Ibáñez et al. [26] observed associations between physical activity levels and somatotype components among orienteering athletes. The findings of the present study are partly consistent with these observations, as adjusted analyses showed that greater walking time was independently associated with a higher mesomorphic component. However, it should be emphasized that the observed associations were weak and were limited to walking time. In contrast, in the primary adjusted analysis of the total sample, moderate- and vigorous-intensity physical activity were not significantly associated with any somatotype component. These findings suggest that the associations between different forms of physical activity and somatotype components require further investigation, particularly among young adult populations.

In the primary adjusted analysis of the total sample, significant associations with somatotype components were observed exclusively for walking time. However, it should be emphasized that the observed associations were weak. It is also noteworthy that walking time was not significantly correlated with somatotype components in the analysis of the total sample, whereas after adjustment for sex, age, and country of origin, it was significantly associated with all three somatotype components. This may suggest that simple associations between walking and body morphology characteristics were partly masked by differences in sex and country of origin, which became apparent after adjustment in the regression models. Sex and country of origin also showed some of the strongest associations with somatotype components in the regression models. Because the Polish and Slovenian participants were recruited from different academic programmes, the association with country may partly reflect differences in educational background and sample composition rather than country-specific characteristics alone. Additional country-stratified analyses identified several statistically significant associations within the Polish and Slovenian samples. Among Slovenian participants, walking time was positively associated with endomorphy and negatively associated with ectomorphy. Moderate-intensity physical activity was also negatively associated with ABSI among Slovenian participants. Among Polish participants, moderate-intensity physical activity was positively associated with endomorphy and negatively associated with ectomorphy. Sleep duration was also negatively associated with mesomorphy among Polish participants. However, because no formal interaction tests between country and behaviour were performed, differences in statistical significance between the country-stratified analyses should not be interpreted as evidence that the associations differed significantly between countries. These findings should be interpreted cautiously, particularly because of the unequal sample sizes and sex distributions between the two country groups, and should not be considered evidence of statistically significant differences between countries. The observed findings are difficult to interpret unequivocally because walking time, recorded using the study-specific activity record, included both recreational walking and walking associated with everyday transportation and daily activities. Consequently, the identified adjusted associations may reflect general patterns of daily mobility rather than a specific relationship between walking and body morphology. Furthermore, due to the cross-sectional design of the study, the direction of the observed associations cannot be determined. The observed associations may operate in either direction; for example, individuals with greater endomorphy may engage in more walking as a compensatory behaviour. However, this possibility cannot be verified in the present cross-sectional study.

In the present study, no significant associations were found between sedentary behaviour and the analysed body morphology indicators. Regarding sleep, only a few weak associations were observed with selected somatotype components. These included associations in the sex-stratified analyses and a negative association between sleep duration and mesomorphy among Polish participants in the country-stratified analysis. No significant association was identified between sleep duration and ABSI. These findings are partly consistent with the observations of Kozak et al. [35], who, in a two-year longitudinal study of university students, found no significant associations between sleep duration and changes in BMI or body fat percentage. However, the authors reported that deteriorating sleep quality was associated with an increase in BMI. In contrast, Borowska et al. [36] and Song et al. [37] demonstrated associations between sleep duration and other sleep-related behaviours and selected indicators of adiposity. Discrepancies between studies may result from differences in population characteristics as well as the methods used to assess sleep and body morphology. In light of the available evidence, it appears that the associations between sleep and body morphology may be more strongly linked to overall sleep quality and sleep patterns than to sleep duration alone, a conclusion also emphasized by Chaput et al. [4]. In the primary adjusted analysis of the total sample, independent associations with the analysed health behaviours were observed only for somatotype components and not for ABSI. However, these associations were few in number and characterized by small effect sizes. Therefore, the results should be interpreted with caution and should be understood as showing different patterns of associations for ABSI and somatotype components within the present sample, rather than indicating the superiority of one measure over the other. The limited strength of the observed associations further suggests that body morphology characteristics in young adults are likely related to multiple interacting factors beyond the health behaviours examined in the present study.

This study has several limitations. First, its cross-sectional design precludes causal inference. Second, physical activity, sedentary behaviour, and sleep duration were assessed using self-reported measures, which are subject to recall bias and reporting inaccuracies. In addition, the participant-applied talk test used to distinguish between moderate- and vigorous-intensity physical activity may have resulted in some misclassification between these intensity categories. Third, the assessment of sleep was limited to duration and did not include measures of sleep quality or regularity. Fourth, the activity record did not allow for a detailed characterization of the types of physical activity performed. Physical activity and sleep behaviours may also vary across seasons, and the timing of data collection may therefore have influenced the observed associations. Furthermore, the analyses did not account for other factors potentially related to body morphology, such as dietary habits, stress levels, or genetic predisposition. Most of the observed associations were exploratory in nature, and as no formal adjustment for multiple comparisons was applied, the large number of correlation analyses performed increases the possibility that some statistically significant findings may have occurred by chance. Furthermore, the observed effect sizes were generally small. Although the achieved sample size allowed the detection of effects of approximately f^2^ = 0.04, smaller associations may not have been detected with adequate statistical power. Therefore, the findings should be interpreted with caution and confirmed in future studies. Finally, the study was conducted among university students from two European countries representing different fields of study, which may have influenced both physical activity levels and body morphology characteristics. Although country of origin was included as a control variable in the primary regression analyses and additional country-stratified analyses were performed, academic programme could not be examined independently because it was closely linked to country. Therefore, potential residual confounding related to differences in academic profile between the Polish and Slovenian samples cannot be excluded. It should also be acknowledged that participants were recruited from specific academic programmes, which may limit the representativeness of the sample and the generalizability of the findings to the broader population of young adults. Despite these limitations, the study provides novel data on the associations between health behaviours and both ABSI and somatotype components in young adults, helping to improve the understanding of how these body morphology indicators relate to everyday movement behaviours in this population.

## 5. Conclusions

In the primary adjusted analysis of the total sample, no significant associations were found between physical activity, sedentary behaviour, sleep duration, and ABSI. Only a few weak associations were observed between selected health behaviours and the somatotype profile, reflected by the combined pattern of its endomorphic, mesomorphic, and ectomorphic components. The observed effect sizes were small, and the regression models explained only a limited proportion of the variance in the analysed body morphology indicators. Country-stratified analyses identified several statistically significant associations within the Polish and Slovenian samples. These findings support a cautious interpretation of the results for the total sample. ABSI and somatotype components also showed different patterns of associations, reflecting the different aspects of body morphology assessed by these measures. This should not be interpreted as evidence of the superiority of either measure. Further research, particularly longitudinal studies using objective measures of physical activity and sleep and incorporating a broader range of factors that may influence body morphology, is warranted.

## Figures and Tables

**Table 1:** Anthropometric Characteristics of the Study Participants.

Variable	Total (*n* = 371)	Men (*n* = 202)	Women (*n* = 169)
Body height (cm)	174.90 ± 9.51	181.04 ± 7.41	167.57 ± 5.85
Body mass (kg)	71.34 ± 14.52	79.72 ± 10.89	61.33 ± 11.72
Biepicondylar humerus breadth (cm)	6.57 ± 0.68	7.00 ± 0.53	6.05 ± 0.42
Biepicondylar femur breadth (cm)	9.42 ± 0.75	9.80 ± 0.62	8.96 ± 0.63
Flexed arm circumference (cm)	31.15 ± 4.14	33.88 ± 2.96	27.88 ± 2.75
Waist circumference (cm)	77.11 ± 9.63	82.48 ± 7.48	70.69 ± 7.82
Hip circumference (cm)	95.95 ± 7.47	95.62 ± 7.28	96.34 ± 7.69
Calf circumference (cm)	36.63 ± 2.90	37.34 ± 2.58	35.78 ± 3.05
Triceps skinfold (mm)	12.02 ± 5.21	9.25 ± 4.05	15.32 ± 4.47
Subscapular skinfold (mm)	11.82 ± 4.60	11.45 ± 3.86	12.27 ± 5.34
Supraspinous skinfold (mm)	12.79 ± 6.07	12.98 ± 6.22	12.56 ± 5.88
Medial calf skinfold (mm)	12.56 ± 5.79	11.14 ± 5.66	14.27 ± 5.49

Data are presented as mean ± standard deviation (SD).

**Table 2:** Body morphology and movement behaviour characteristics of the study participants.

Variable	Total (*n* = 371)	Men (*n* = 202)	Women (*n* = 169)
Age (years)	21.46 ± 1.07	21.54 ± 1.05	21.37 ± 1.08
BMI (kg/m^2^)	23.16 ± 3.40	24.30 ± 2.81	21.80 ± 3.56
WHR	0.80 ± 0.09	0.86 ± 0.07	0.73 ± 0.05
WHtR	0.44 ± 0.05	0.46 ± 0.04	0.42 ± 0.05
ABSI	0.074 ± 0.004	0.077 ± 0.003	0.071 ± 0.003
Endomorphy	3.59 ± 1.24	3.18 ± 1.08	4.08 ± 1.24
Mesomorphy	4.21 ± 1.24	4.71 ± 1.01	3.61 ± 1.23
Ectomorphy	2.54 ± 1.17	2.36 ± 1.07	2.74 ± 1.24
Walking (min/week)	200 (70–360)	150 (60–270)	280 (120–450)
Moderate PA (min/week)	150 (90–280)	150 (90–270)	180 (90–300)
Vigorous PA (min/week)	90 (60–150)	90 (60–150)	90 (60–180)
Sitting (h/week)	48 (42–58)	49 (42–58)	48 (42–56)
Sleep (h/week)	52 (50–55)	52 (50–55)	52 (49–55)

Data are presented as mean ± standard deviation (SD) or median (interquartile range, IQR; Q1–Q3). BMI—body mass index; WHR—waist-to-hip ratio; WHtR—waist-to-height ratio; ABSI—A Body Shape Index; PA—physical activity.

**Table 3:** Spearman’s rank correlations between physical activity, sedentary behaviour, sleep duration, ABSI, and somatotype components.

Variable	ABSI	Endomorphy	Mesomorphy	Ectomorphy
Total sample (*n* = 371)				
Walking (min/week)	−0.135 **	0.083	−0.073	−0.020
Moderate PA (min/week)	−0.080	0.060	0.031	−0.099
Vigorous PA (min/week)	−0.071	−0.052	−0.013	0.043
Sitting (h/week)	0.013	−0.050	0.010	−0.024
Sleep (h/week)	0.036	−0.062	−0.111 *	0.098
Men (*n* = 202)				
Walking (min/week)	−0.056	−0.076	0.094	−0.059
Moderate PA (min/week)	−0.060	0.052	−0.033	−0.086
Vigorous PA (min/week)	0.012	−0.049	0.008	0.024
Sitting (h/week)	−0.061	−0.004	0.008	−0.138 *
Sleep (h/week)	−0.005	−0.010	−0.092	0.032
Women (*n* = 169)				
Walking (min/week)	−0.068	0.041	−0.017	−0.049
Moderate PA (min/week)	−0.147	0.068	0.122	−0.154 *
Vigorous PA (min/week)	−0.162 *	−0.118	0.090	0.033
Sitting (h/week)	0.072	−0.082	−0.042	0.125
Sleep (h/week)	0.062	−0.085	−0.194 *	0.166 *

ABSI—A Body Shape Index; PA—physical activity; * *p* < 0.05; ** *p* < 0.01.

**Table 4:** Multiple linear regression models examining associations of physical activity, sedentary behaviour, and sleep with ABSI and somatotype components.

A. ABSI × 1000; R^2^ = 0.106; Adjusted R^2^ = 0.086; F = 5.34; *p* < 0.001					
Independent variable	B	SE	β	95% CI	*p*
Walking (100 min/week)	−0.022	0.126	−0.011	−0.270 to 0.225	0.859
Moderate PA (100 min/week)	−0.166	0.183	−0.046	−0.525 to 0.193	0.363
Vigorous PA (100 min/week)	−0.256	0.222	−0.060	−0.692 to 0.179	0.248
Sitting (h/week)	0.004	0.023	0.009	−0.042 to 0.050	0.858
Sleep (h/week)	0.025	0.054	0.023	−0.082 to 0.132	0.648
Sex	−2.599	0.554	−0.261	−3.690 to −1.509	<0.001
Age (years)	−0.026	0.234	−0.006	−0.485 to 0.433	0.911
Country	0.905	0.713	0.083	−0.497 to 2.307	0.205
B. Endomorphy; R^2^ = 0.156; Adjusted R^2^ = 0.137; F = 8.36; *p* < 0.001					
Independent variable	B	SE	β	95% CI	*p*
Walking (100 min/week)	0.081	0.031	0.158	0.021 to 0.141	0.009
Moderate PA (100 min/week)	0.034	0.044	0.038	−0.053 to 0.121	0.444
Vigorous PA (100 min/week)	−0.018	0.054	−0.017	−0.123 to 0.088	0.743
Sitting (h/week)	−0.004	0.006	−0.034	−0.015 to 0.007	0.491
Sleep (h/week)	−0.009	0.013	−0.034	−0.035 to 0.017	0.485
Sex	0.942	0.134	0.379	0.677 to 1.206	<0.001
Age (years)	0.063	0.057	0.054	−0.048 to 0.175	0.266
Country	0.354	0.173	0.130	0.014 to 0.694	0.041
C. Mesomorphy; R^2^ = 0.220; Adjusted R^2^ = 0.203; F = 12.77; *p* < 0.001					
Independent variable	B	SE	β	95% CI	*p*
Walking (100 min/week)	0.060	0.029	0.117	0.002 to 0.118	0.041
Moderate PA (100 min/week)	0.000	0.043	0.000	−0.084 to 0.084	0.999
Vigorous PA (100 min/week)	0.030	0.052	0.028	−0.072 to 0.132	0.563
Sitting (h/week)	−0.003	0.005	−0.028	−0.014 to 0.008	0.558
Sleep (h/week)	−0.024	0.013	−0.087	−0.049 to 0.001	0.064
Sex	−1.055	0.129	−0.424	−1.310 to −0.801	<0.001
Age (years)	−0.080	0.055	−0.069	−0.188 to 0.027	0.141
Country	0.364	0.166	0.134	0.037 to 0.692	0.029
D. Ectomorphy; R^2^ = 0.086; Adjusted R^2^ = 0.065; F = 4.24; *p* < 0.001					
Independent variable	B	SE	β	95% CI	*p*
Walking (100 min/week)	−0.093	0.030	−0.192	−0.152 to −0.034	0.002
Moderate PA (100 min/week)	−0.065	0.043	−0.076	−0.150 to 0.021	0.137
Vigorous PA (100 min/week)	0.010	0.053	0.010	−0.094 to 0.114	0.852
Sitting (h/week)	−0.002	0.006	−0.020	−0.013 to 0.009	0.694
Sleep (h/week)	0.021	0.013	0.080	−0.005 to 0.046	0.113
Sex	0.258	0.132	0.110	−0.002 to 0.518	0.052
Age (years)	0.059	0.056	0.054	−0.051 to 0.168	0.291
Country	−0.657	0.170	−0.255	−0.991 to −0.323	<0.001

ABSI values were multiplied by 1000 for presentation in panel A. B—unstandardized regression coefficient; SE—standard error; β—standardized regression coefficient; CI—confidence interval; R^2^—coefficient of determination; Adjusted R^2^—adjusted coefficient of determination. PA—physical activity. Physical activity variables are expressed per 100 min/week; sitting and sleep are expressed in h/week.

**Table 5:** Country-stratified multiple linear regression analyses of associations between lifestyle behaviours and body morphology indicators.

A—Slovenia (n 108): ABSI: R^2^ = 0.194, adjusted R^2^ = 0.137, model *p* = 0.002; Endomorphy: R^2^ = 0.189, adjusted R^2^ = 0.132, model *p* = 0.003; Mesomorphy: R^2^ = 0.099, adjusted R^2^ = 0.036, model *p* = 0.155; Ectomorphy: R^2^ = 0.087, adjusted R^2^ = 0.023, model *p* = 0.228.					
Dependent variable	Independent variable	B	95% CI	β	*p*
ABSI × 1000	Walking time	−0.057	−0.249 to 0.135	−0.054	0.557
	Moderate PA	−0.556	−0.952 to −0.161	−0.269	0.006
	Vigorous PA	−0.192	−0.650 to 0.266	−0.082	0.408
	Sitting time	0.012	−0.037 to 0.061	0.047	0.628
	Sleep duration	−0.026	−0.127 to 0.076	−0.047	0.615
Endomorphy	Walking time	0.100	0.017 to 0.183	0.221	0.019
	Moderate PA	−0.085	−0.256 to 0.087	−0.095	0.330
	Vigorous PA	0.034	−0.164 to 0.233	0.034	0.731
	Sitting time	−0.001	−0.022 to 0.020	−0.009	0.930
	Sleep duration	−0.028	−0.071 to 0.016	−0.117	0.217
Mesomorphy	Walking time	0.058	−0.030 to 0.146	0.128	0.194
	Moderate PA	−0.057	−0.239 to 0.124	−0.064	0.532
	Vigorous PA	0.096	−0.114 to 0.306	0.095	0.368
	Sitting time	−0.002	−0.025 to 0.020	−0.022	0.828
	Sleep duration	−0.008	−0.055 to 0.038	−0.034	0.730
Ectomorphy	Walking time	−0.091	−0.173 to −0.008	−0.214	0.032
	Moderate PA	0.032	−0.138 to 0.202	0.039	0.708
	Vigorous PA	−0.097	−0.294 to 0.100	−0.103	0.330
	Sitting time	0.003	−0.019 to 0.024	0.025	0.810
	Sleep duration	0.026	−0.018 to 0.070	0.118	0.240
B—Poland (n 263): ABSI: R^2^ = 0.063, adjusted R^2^ = 0.037, model *p* = 0.020; Endomorphy: R^2^ = 0.165, adjusted R^2^ = 0.142, model *p* < 0.001; Mesomorphy: R^2^ = 0.230, adjusted R^2^ = 0.208, model *p* < 0.001; Ectomorphy: R^2^ = 0.057, adjusted R^2^ = 0.031, model *p* = 0.034.					
Dependent variable	Independent variable	B	95% CI	β	*p*
ABSI × 1000	Walking time	−0.014	−0.554 to 0.526	−0.003	0.959
	Moderate PA	0.137	−0.380 to 0.653	0.032	0.603
	Vigorous PA	−0.201	−0.836 to 0.435	−0.038	0.534
	Sitting time	−0.007	−0.073 to 0.058	−0.013	0.829
	Sleep duration	0.108	−0.064 to 0.280	0.075	0.218
Endomorphy	Walking time	0.005	−0.103 to 0.112	0.005	0.933
	Moderate PA	0.123	0.020 to 0.226	0.137	0.020
	Vigorous PA	−0.027	−0.153 to 0.100	−0.024	0.680
	Sitting time	−0.007	−0.020 to 0.006	−0.063	0.283
	Sleep duration	0.020	−0.014 to 0.054	0.066	0.256
Mesomorphy	Walking time	0.065	−0.034 to 0.164	0.071	0.199
	Moderate PA	0.014	−0.081 to 0.109	0.016	0.770
	Vigorous PA	−0.008	−0.124 to 0.109	−0.007	0.894
	Sitting time	−0.003	−0.015 to 0.009	−0.031	0.586
	Sleep duration	−0.037	−0.069 to −0.006	−0.129	0.020
Ectomorphy	Walking time	−0.087	−0.193 to 0.020	−0.098	0.109
	Moderate PA	−0.104	−0.205 to −0.002	−0.125	0.046
	Vigorous PA	0.076	−0.049 to 0.201	0.074	0.233
	Sitting time	−0.006	−0.019 to 0.007	−0.055	0.376
	Sleep duration	0.009	−0.025 to 0.042	0.031	0.616

B, unstandardized regression coefficient; β, standardized regression coefficient; CI, confidence interval; PA, physical activity; ABSI, A Body Shape Index. Models were adjusted for sex and age. Unstandardized coefficients for walking, moderate-intensity physical activity, and vigorous-intensity physical activity represent the change in the dependent variable per 100 min/week. Sitting time and sleep duration are expressed in h/week. ABSI values were multiplied by 1000 to improve the readability of the regression coefficients.

## Data Availability

The data supporting the findings of this study are available from the corresponding author upon reasonable request. The data are not publicly available due to ethical and privacy restrictions.
